# Genome Sequence of a Clostridioides difficile Strain Isolated from Feces from a Patient in Southern Thailand

**DOI:** 10.1128/mra.00455-23

**Published:** 2023-06-15

**Authors:** Thunchanok Yaikhan, Komwit Surachat, Chonticha Romyasamit, Pawarisa Luenglusontigit, Arnon Chukamnerd, Phanvasri Saengsuwan, Monwadee Wonglapsuwan, Kamonnut Singkhamanan

**Affiliations:** a Department of Biomedical Sciences and Biomedical Engineering, Faculty of Medicine, Prince of Songkla University, Songkhla, Thailand; b Translational Medicine Research Center, Faculty of Medicine, Prince of Songkla University, Songkhla, Thailand; c Department of Medical Technology, School of Allied Health Sciences, Walailak University, Nakhon Si Thammarat, Thailand; d Division of Infectious Diseases, Department of Internal Medicine, Faculty of Medicine, Prince of Songkla University, Songkhla, Thailand; e Division of Biological Science, Faculty of Science, Prince of Songkla University, Songkhla, Thailand; University of Rochester School of Medicine and Dentistry

## Abstract

Clostridioides difficile is a Gram-positive, obligate anaerobic, toxin-producing bacillus that is linked to antibiotic-associated diarrhea. Here, we report the whole-genome sequence of a C. difficile strain isolated from stool from a patient, using next-generation sequencing (MGISEG-2000). *De novo* assembly revealed a genome length of 4,208,266 bp. Multilocus sequence typing (MLST) results showed that the isolate belonged to sequence type 23 (ST23).

## ANNOUNCEMENT

**FIG 1:**
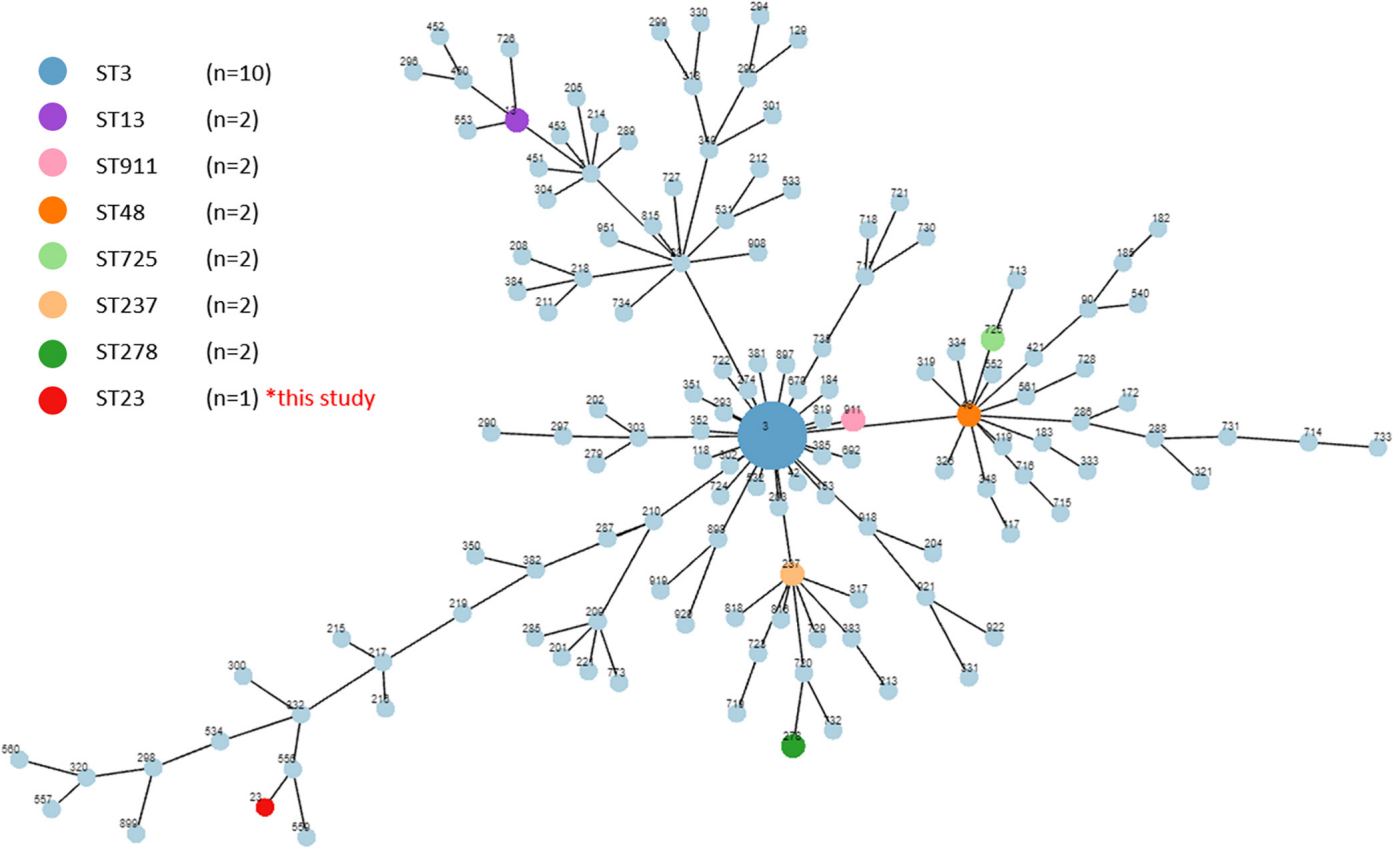
MSTree of C. difficile isolates found in Asian countries, based on MLST using seven genes of C. difficile. Each node in the tree represents a ST, and the size of the node proportionally represents the number of isolates.

Clostridioides difficile is a toxin-producing bacterium that is considered the etiological pathogen of nosocomial antibiotic-associated diarrhea (1). The excessive or unindicated use of antibiotics probably results in a disturbance of the normal flora in the colon that leads to increased growth of pathogenic C. difficile, which can produce toxins that trigger pseudomembranous colitis (2). Genotypic analyses may not be able to comprehensively detect all genes associated with the severity of an illness. Whole-genome sequencing (WGS) may identify genes that can contribute to comprehensive genome information, such as antimicrobial resistance genes, mobile genetic elements, and virulence-associated genes. In this work, we report the whole-genome sequence of a C. difficile strain that was isolated from a patient with clinical signs consistent with C. difficile infection. The isolate was collected from the stool of a patient in Songklanagarind Hospital, in southern Thailand, in 2014. The isolate was cultured on cycloserine-cefoxitin-fructose agar at 37°C for 72 h under anaerobic conditions, and a single large colony with a creamy yellow to gray-white color was collected for bacterial DNA extraction. The colonies on the plate were preserved in 30% glycerol in brain heart infusion broth at −80°C for further analyses. The genomic DNA of the bacterium was extracted using the QIAamp DNA minikit (Qiagen, Germany), according to the manufacturer’s instructions. The DNA concentration and purification were determined using NanoDrop 2000/2000c spectrophotometers (Thermo Fisher Scientific), and the size and degradation of the DNA were determined by agarose gel electrophoresis. For DNA sequencing, DNA was sent to the company for library preparation, and DNA libraries were subjected to short-read sequencing with the MGISEQ-2000 system (BGI, Beijing, China) to generate 150-bp paired-end reads. Raw sequence reads were checked for quality with FastQC (3), and then low-quality reads and sequence adapters were removed using fastp (4). Next, filtered reads were *de novo* assembled using SPAdes (5). The assembled sequences were qualitatively checked by aligning contigs to the reference genome using QUAST (6). The completeness evaluation of the genome assembly was performed with the BUSCO pipeline by offering biologically relevant metrics based on expected gene content (7). The average nucleotide identity (ANI) was computed according to the alignment-free algorithm of FastANI v1.32 (8). All analysis software was set to default parameters. The *de novo* genome analysis reported a total of 39 contigs, with an *N*_50_ value of 246,284 bp. The taxonomic classification annotated the isolate as C. difficile on the basis of an ANI value of 97.96%, compared with reference C. difficile strain S-0253 (GenBank accession number NZ_CP076401). Our isolate showed a whole-genome length of 4,208,266 bp, with 100× coverage, and the GC content of the sequences was 33%. The number of coding sequences (CDSs) was 3,636, which accounted for 97.5% of the coding length. The numbers of rRNAs and tRNAs were 56 and 43, respectively. Multilocus sequence typing (MLST) assigned the C. difficile strain to sequence type 23 (ST23) on the basis of seven housekeeping genes, namely, *adk*, *atpA*, *dxr*, *glyA*, *recA*, *sodA*, and *tpi*, and the ST was identified using the PubMLST database (9). For the correlation between the C. difficile strain in this study and other isolates from Asian countries, a minimum spanning tree (MSTree) was generated using PHYLOViZ 2.0 based on the ST; the analysis was performed using default parameters (10). The MSTree analysis of MLST data for 152 C. difficile isolates, representing 130 STs, is illustrated in Fig. 1. This announcement provides whole-genome information on C. difficile and its ST, which might assist in understanding the pathogen causing pseudomembranous colitis.

This clinical research study was approved by the Human Research Ethics Committee (HREC) of the Faculty of Medicine, Prince of Songkla University, under certificates of approval REC.57-0150-04-2 and REC.64-550-4-2.

### Data availability.

The complete genome sequence has been deposited in GenBank under BioProject accession number PRJNA943619, BioSample accession number SAMN33732448, SRA accession number SRR23867102, and genome accession number JARJHO000000000.
